# Differential Effects of 670 and 830 nm Red near Infrared Irradiation Therapy: A Comparative Study of Optic Nerve Injury, Retinal Degeneration, Traumatic Brain and Spinal Cord Injury

**DOI:** 10.1371/journal.pone.0104565

**Published:** 2014-08-08

**Authors:** Marcus K. Giacci, Lachlan Wheeler, Sarah Lovett, Emma Dishington, Bernadette Majda, Carole A. Bartlett, Emma Thornton, Elizabeth Harford-Wright, Anna Leonard, Robert Vink, Alan R. Harvey, Jan Provis, Sarah A. Dunlop, Nathan S. Hart, Stuart Hodgetts, Riccardo Natoli, Corinna Van Den Heuvel, Melinda Fitzgerald

**Affiliations:** 1 Experimental and Regenerative Neurosciences, The University of Western Australia, Crawley, Australia; 2 School of Animal Biology, The University of Western Australia, Crawley, Australia; 3 School of Anatomy, Physiology and Human Biology, The University of Western Australia, Crawley, Australia; 4 School of Medical Sciences, The University of Adelaide, Adelaide, Australia; 5 ANU Medical School and John Curtin School of Medical Research, The Australian National University, Canberra, Australia; 6 Neuroecology Group, The Oceans Institute, The University of Western Australia, Crawley, Australia; University of South Florida, United States of America

## Abstract

Red/near-infrared irradiation therapy (R/NIR-IT) delivered by laser or light-emitting diode (LED) has improved functional outcomes in a range of CNS injuries. However, translation of R/NIR-IT to the clinic for treatment of neurotrauma has been hampered by lack of comparative information regarding the degree of penetration of the delivered irradiation to the injury site and the optimal treatment parameters for different CNS injuries. We compared the treatment efficacy of R/NIR-IT at 670 nm and 830 nm, provided by narrow-band LED arrays adjusted to produce equal irradiance, in four *in vivo* rat models of CNS injury: partial optic nerve transection, light-induced retinal degeneration, traumatic brain injury (TBI) and spinal cord injury (SCI). The number of photons of 670 nm or 830 nm light reaching the SCI injury site was 6.6% and 11.3% of emitted light respectively. Treatment of rats with 670 nm R/NIR-IT following partial optic nerve transection significantly increased the number of visual responses at 7 days after injury (P≤0.05); 830 nm R/NIR-IT was partially effective. 670 nm R/NIR-IT also significantly reduced reactive species and both 670 nm and 830 nm R/NIR-IT reduced hydroxynonenal immunoreactivity (P≤0.05) in this model. Pre-treatment of light-induced retinal degeneration with 670 nm R/NIR-IT significantly reduced the number of Tunel+ cells and 8-hydroxyguanosine immunoreactivity (P≤0.05); outcomes in 830 nm R/NIR-IT treated animals were not significantly different to controls. Treatment of fluid-percussion TBI with 670 nm or 830 nm R/NIR-IT did not result in improvements in motor or sensory function or lesion size at 7 days (P>0.05). Similarly, treatment of contusive SCI with 670 nm or 830 nm R/NIR-IT did not result in significant improvements in functional recovery or reduced cyst size at 28 days (P>0.05). Outcomes from this comparative study indicate that it will be necessary to optimise delivery devices, wavelength, intensity and duration of R/NIR-IT individually for different CNS injury types.

## Introduction

Oxidative stress occurs when the production of reactive oxygen (ROS) and nitrogen (RNS) species overwhelms the endogenous antioxidant reducing enzymes, and is considered a hallmark of injury to the central nervous system (CNS) [1]–[6]. The high rate of oxidative metabolic activity and associated reactive oxygen metabolites, low antioxidant capacity, and high content of polyunsaturated fats render CNS neurons and glia particularly prone to the deleterious effects of oxidative stress. Furthermore, during secondary degeneration, mitochondrial dysfunction and oxidative stress occur soon after injury [4], [7], [8]. As such, the alleviation of oxidative stress is an important therapeutic strategy for the treatment of neurotrauma. However, despite encouraging pre-clinical assessments of numerous antioxidants, there are currently no effective antioxidant strategies for attenuation of ROS production in clinical use following neurotrauma [9].

Irradiation in the red/near infrared spectrum (R/NIR, 630–1000 nm) has been developed as a therapeutic strategy to treat a range of injuries and diseases. Improvements following R/NIR-IT have been reported both in animals and humans in a wide range of injuries and disease including cardial infarct [10], renal and hepatic complications during diabetes [11], [12], and oral mucositis [13]. In addition, conditions specific to the nervous system have shown improved recovery following R/NIR-IT, including retinal degeneration [14], [15], CNS injury and stroke [16]–[22]. Clinical trials are currently underway assessing R/NIR-IT for stroke, with NeuroThera Effectiveness and Safety Trial (NEST)-1 and NEST-2 already completed with some clinical improvement shown [23], [24]. This has led to the commencement of a third trial (NEST-3). However, the trial was halted following an interim futility analysis [25], perhaps due to a failure to employ effective treatment parameters [17].

While there is substantial controversy regarding the mechanism of action of R/NIR-IT, one widely supported hypothesis is that it acts by improving oxidative metabolism and reducing oxidative stress. The enzyme cytochrome *c* oxidase, complex IV of the electron transport chain, is proposed to act as a photoacceptor for irradiation at R/NIR wavelengths, with peaks in its absorption spectrum matching known efficacious treatment wavelengths. Irradiation is thought to lead to improvements in oxidative metabolism *via* changes in the oxidation-reduction state of the enzyme [26], [27]. Increases in cytochrome *c* oxidase activity with R/NIR-IT are associated with increases in adenosine triphosphate (ATP) content and oxygen consumption *in vitro* and *in vivo*, indicating increased flux through the electron transport chain [28]–[30]. This may lead to lower concentrations of reactive species and reduced oxidative stress, due to maintenance of the mitochondrial permeability transition [31].

Other potential mechanisms of action of R/NIR-IT have been suggested, including effects downstream of cytochrome *c* oxidase activation. Photo-activation of the chromophores haemoglobin, myoglobin, flavins and metal free porphyrins may play a role *via* as yet unknown mechanisms [32]–[34]. Nitric oxide catalysed from cytochrome *c* oxidase may lead to downstream vasodilatation [35], [36] and signal transduction, potentially contributing to functional improvements. R/NIR-IT has also been shown to modulate gene expression [14], reduce apoptosis [29], [37], alter cytokine release and modulate immune responses [20], [38]–[40]; outcomes perhaps independent of modulation of cytochrome *c* oxidase activity. Nevertheless, R/NIR-IT has been shown to reduce indicators of oxidative stress following CNS injury *in vivo* [8], [22], and may serve as an effective first line antioxidant as part of a combinatorial strategy to reduce oxidative stress following neurotrauma.

R/NIR-IT is usually delivered using either lasers or light-emitting diodes (LEDs). LEDs are an increasingly accepted source for R/NIR-IT [41]. It was originally thought that the coherence of laser light was essential to achieve the therapeutic effects of R/NIR-IT, resulting in the widespread use of HeNe lasers (which emit light at a wavelength of 632.8 nm) [41]. More recently, therapeutic benefits of R/NIR-IT have been observed using non-coherent light sources such as LEDs [37], [42], [43], giving credence to the concept of using cheaper and more convenient LED devices to administer treatment. However, very few studies have reported the efficacy of R/NIR-IT delivered by LED for treatment of TBI or SCI *in vivo*. Furthermore, few studies have quantitatively compared the therapeutic effects of different wavelengths of light [20], [29], [44]–[47] and, to our knowledge, none of these compared different wavelengths of R/NIR-IT, delivered by LED, in more than one *in vivo* model of CNS injury. The therapeutic dose can be defined by the number of photons interacting with the photoacceptor (chromophore). As the energy of a photon varies with its wavelength, intensities of irradiation can be adjusted to ensure delivery of equal numbers of photons to provide an effective comparison of the efficacy of R/NIR-IT of different wavelengths [48]. We have conducted a multi-centre study designed to compare efficacy of equal numbers of photons of 670 nm and 830 nm R/NIR-IT, delivered to the surface of the skin overlying injury sites using LED arrays, in four models of CNS injury, each of mild to moderate severity: 1) in a model of secondary degeneration following partial injury to the optic nerve; 2) in light-induced retinal degeneration; 3) in traumatic brain injury (TBI) induced by fluid percussion impact; and 4) following a moderate contusion spinal cord injury (SCI). The R/NIR-IT treatment parameters were kept constant for the three traumatic injury models. Because previous experiments had established the approximate parameters for effective treatment of the retina, a 10-fold lower dose, delivered prior to light damage, was used in the retinal degeneration model. Penetrance of R/NIR-IT to the eye is greater than to the other CNS injury sites [22]. As such, delivery of the full dose to the retina that was used in the other CNS injury models would have resulted in unnecessary exposure to excess irradiation. We have already determined that there is no benefit in delivering this tenfold greater dose in the retinal degeneration model (unpublished). Although we delivered equal numbers of photons of each light source (670 and 830 nm) to the surface of the body, it is possible that any differences in the spectral transmission properties of the intervening tissue(s) could affect the relative irradiance actually received at the injury site. However, given the difficulties in measuring these transmission properties *in vivo*, especially in thick highly-scattering tissues, we attempted to equate quantal irradiance as far as practicable. We have assessed treatment outcomes of behavioural function or neuroprotection, of lesion volume, and of oxidative stress, as appropriate for each model system, and demonstrated differential effects dependent on wavelength used and injury type.

## Materials and Methods

All procedures involving animals complied with the “Principles of Laboratory Animal Care” (National Institutes of Health publication no. 86–23, revised 1985) and were approved by the relevant institutional Animal Ethics Committee (The University of Western Australia approvals: RA3/100/673, RA3/100/1247; Australian National University approval A2011/029; University of Adelaide approval M-2013-160).

### R/NIR-IT delivery, calibration and treatment

The spectral transmittance (300–890 nm) of an excised block of skin and muscle (plus the skin and muscle separately for information purposes) lying dorsal to the spinal column of a Fischer rat cadaver was measured to assess the degree to which light penetrates to the site of injury in the contusion SCI rat model. Broadband ‘white’ light from a 175 W xenon lamp (Spectral Products, CT, USA) was delivered to the dorsal surface of excised tissue via a 600 µm diameter quartz fibre optic fitted with a quartz collimating lens. Light transmitted by the tissue was collected via a cosine-corrector (CC-3-UV, Ocean Optics, Dunedin, FL, USA; covered with a thin layer of ‘cling-wrap’) connected to a 1000 µm diameter quartz fibre optic that delivered the light to an Ocean Optics USB4000 CCD spectroradiometer. Data acquisition was controlled through SpectraSuite software (Ocean Optics). The irradiance of R/NIR-IT received at the spinal cord was calculated by multiplying the spectral irradiances produced by the 670 and 830 nm devices at a surface of the skin, by the measured spectral transmittance of the skin and muscle.

R/NIR-IT was delivered to live rats using two different LED arrays: 670 nm R/NIR-IT from a commercially available device (VET75, Quantum Devices Inc, Barneveld, WI, USA) or 830 nm R/NIR-IT from a custom built device. The custom built adjustable intensity 830 nm LED array was constructed from infra-red LEDS and had a similar emission cross sectional area to the Vet75 device (70 cm^2^). The light output of the custom built 830 nm LED array was adjusted to produce an equal photon irradiance (5.33×10^16^ photons cm^−2^ s^−1^) to the VET75 670 nm LED array, at the surface of the skin (3 cm from the LED array). Because of the difference in photon energy at these two wavelengths, this equal quantal dose equates to an energy dose of 28.4 J cm^−2^ for the 670 nm array and 22.6 J cm^−2^ for the 830 nm array. Treatment of all rats with R/NIR-IT delivered by LED array was conducted using consistent procedures for each model of CNS injury. Rats were either gently held by hand or within a clear perspex box such that the LED arrays delivered irradiation at a distance of 3 cm above the surface of the skin overlying the injury, or 2.5 cm from the eye for the retinal degeneration model. Rats were conscious throughout treatment except for the first treatment after surgery, for which they were still anaesthetised. For the traumatic injury models, treatment duration was 30 minutes, delivered once per day, commencing immediately following surgical injury and continuing until euthanasia; control, sham-treated rats were similarly handled, but the overlying LED array was not turned on (referred to as control). For the retinal degeneration model, treatment duration was 3 minutes (resulting in lower doses of 3.4 J cm^−2^ for the 670 nm array or 2.7 J cm^−2^ for the 830 nm array at the eye, in this model) and occurred daily, for the 5 days prior to 24 hours' exposure to bright light.

### Partial optic nerve injury animals and surgery

Adult, female Piebald-Virol-Glaxo (PVG) hooded rats were procured from the Animal Resources Centre (Murdoch, WA), and housed under temperature controlled conditions on a 12 hour light/dark cycle, with access to standard rat chow and water *ad libitum*. The partial optic nerve transection procedure was conducted as described elsewhere [4]. Briefly, PVG rats were anaesthetized (i.p. injection of 50 mg/kg ketamine hydrochloride and 10 mg/kg xylazil hydrochloride, Troy Laboratories, NSW, Australia), the right optic nerve exposed surgically and a diamond keratotomy knife (Geuder, Germany) used to make a controlled dorsal incision in each ON, to a depth of 200 µm. Post-operative analgesia was provided once (subcutaneous injection of 2.8 mg/kg Carprofen; Norbrook Australia, Pty. Ltd., VIC, Australia). Each treatment or control group consisted of 12 rats, and the groups were control, 670 nm or 830 nm R/NIR-IT treated, i.e. a total of 36 rats for this model, all animals experienced partial optic nerve transection.

Optokinetic nystagmus visual reflex was assessed in all rats 7 days after injury and daily R/NIR-IT using established procedures [22], by an investigator blinded to animal identity and treatment. Following behavioural assessments, under anaesthesia as described above, the injured optic nerves in 6 rats/treatment group were dissected from the ocular cavity, collected onto a microscope slide maintained at −20°C over a bed of dry ice, mounted in O.C.T. (optical cutting temperature compound) (Tissue-Tek, Sakura, Japan) then snap-frozen in eppendorf tubes in liquid nitrogen. Rats were then euthanased with Lethabarb© (800–1000 mg/kg i.p, Virbac, Australia Pty. Ltd., NSW, Australia). Immediately following euthanasia, the remaining 6 rats per treatment group were transcardially perfused with 0.9% saline followed by 4% paraformaldehyde (PFA) (0.1 M phosphate buffer; pH 7.2) for immunohistochemical assessments of optic nerves.

### Partial optic nerve injury outcome measures

Fresh-frozen optic nerves were cryosectioned longitudinally at −20°C and free floating tissue sections (20 µm thickness) from each rat were labelled with 10 µM dihydroethidium (DHE) (Life Technologies, VIC, Australia) in phosphate buffered saline (PBS) for 10 minutes, for detection of reactive species. Optic nerves for immunohistochemical analyses were cryosectioned and processed according to established procedures [4], using primary antibodies recognising: manganese superoxide dismutase (MnSOD) (1∶500, Stressgen); heme oxygenase-1 (HO-1) (1∶200; Abcam); glutathione peroxidase 1 (GPx1) (1∶250; Abcam); 3-nitrotyrosine (3NT) (1∶500; Abcam); 4-hydroxynonenal (HNE) (1∶200; Jomar Bioscience); 8-hydroxyguanosine (8OHDG) (1∶500; Abcam); NG2 (1∶250, Invitrogen) and olig2 (1∶500, Abcam) to recognise oligodendrocyte precursor cells as well as Hoechst nuclear stain (1∶1000; Invitrogen, VIC, Australia). Secondary antibodies were species-specific AlexaFluor 555- and 488-conjugated antibodies (1∶500; Invitrogen, VIC, Australia).

Reactive species and immunohistochemical labelling were visualized in a single section of ventral optic nerve directly below the primary injury site for each animal, and photographed using a Leitz Diaplan fluorescence microscope (Leica, Germany). All images for each outcome measure were captured at constant exposures and in a single session. Image analysis was conducted on a single image using Image J/Fiji analysis software, setting constant arbitrary threshold intensities for all images in an analysis and semi-quantifying mean intensities and areas above that threshold. Data shown are either mean areas or intensities above threshold, with choice of analysis dependent on the pattern of staining and the most pronounced increases observed with injury compared to normal optic nerve (unpublished data).

### Retinal degeneration animals and light damage

Albino Sprague-Dawley rats were born and reared in dim cyclic light conditions (12 hr:12 hr, light:dark) with an ambient light level of approximately 5 lux. Between post-natal days (P) 90–120, and following 5 days of R/NIR-IT pre-treatment, photochemical damage to the retina (light damage, LD) was induced in some groups of rats by 24 hours' exposure to a cold-white fluorescent light source positioned above the cages (18 W, Cool White; TFC), at an intensity of approximately 1000 lux at the cage floor. The six treatment or control groups consisted of 6 rats (n = 6), - control, 670 nm or 830 nm R/NIR-IT treated, LD, 670 nm+LD and 830 nm+LD - a total of 36 rats. At the end of the LD period, rats were euthanized with an overdose of barbiturate (60 mg/kg bodyweight i.p., Valabarb; Virbac, Milperra, Australia).

### Retinal degeneration outcome measures

The eyes from the animals was marked at the superior surface for orientation, enucleated and then processed for cryosectioning by immersion fixation in 4% PFA in 0.1 M PBS (pH 7.3) for 3 h at room temperature. The fixed eyes were then washed in PBS (3×5 minutes) and placed in 30% sucrose overnight. Eyes were oriented and embedded in O.C.T. compound, snap frozen in dry ice cooled acetone and cryosectioned at 16 µm. The sections were mounted on superfrost-plus glass slides and dried overnight at 37°C. Sections were stained for histological examined using either immunohistochemistry or TUNEL.

TUNEL staining was used to quantify photoreceptor apoptosis/death following LD in frozen sections using our published method [49]. Counts of TUNEL positive cells in the outer nuclear layer (ONL) were carried out along the retinal sections cut in the para-sagittal plane (superoinferior), including the optic disc. The final count from each animal was then averaged at comparable locations in two non-sequential sections. Sections adjacent, or close, to those used for TUNEL analysis were used for immunohistochemistry. Sections were rehydrated in graded ethanols and then placed in 10% normal goat serum (Sigma) for 1 h to block non-specific binding. Primary antibodies were GFAP (1∶700 Dako); and 8OHDG (1∶500; Abcam) incubated overnight at 4°C. Secondary antibodies were anti-mouse IgG-alexa 594 or anti-rabbit IgG-alexa 488 (1∶1000, Life Technologies) incubated overnight at 4°C. Sections were washed in 0.1 M PBS and coverslipped in a ProLong Gold Antifade (Life Technologies). Primary antibodies were omitted for controls assessing non-specific binding of the secondary antibody. Images were visualised and captured using a LSM5 Confocal microscope (Zeiss), Pascal (Zeiss, v4.0) and prepared using Photoshop CS5 (Adobe). No manipulation to the intensity or gain was performed using either software.

### TBI animals and surgery

A total of 21 adult male Sprague-Dawley rats (weighing between 300–330 grams) were randomly assigned into 4 groups: sham uninjured animals (n = 5), injured untreated controls (n = 6), 670 nm R/NIR-IT treated (n = 5), 830 nm R/NIR-IT treated (n = 5). To administer TBI (n = 16) we used the lateral fluid percussion model (LFP), which has been co-developed by our laboratory in collaboration with others [50] This focal model of TBI produces extensive oxidative stress, inflammation, ion changes, mitochondrial damage and energy crisis, along with both motor and cognitive deficits [51]. Briefly, rats were obtained one to two weeks prior to injury and group housed with unrestricted access to food and water. On the day of trauma, rats were initially anaesthetised via nose cone induction by inhalation of 3–4% isoflurane in 1.5 L/min O_2_, subsequently intubated and then mechanically ventilated to a surgical level by inhalation of 1–2% isoflurane in 1.5 L/min O_2_.

Under anaesthesia, a midline incision was made on the skin overlaying the skull and the bone exposed. Lignocaine (local anaesthetic) was injected prior to incision. After exposing the skull, a 4 mm craniotomy centered over the parietal cortex between the midline and the temporal ridge was performed using a trephine drill. Care was taken not to damage the dura which was left intact at the opening. A female luer loc was then cemented over the craniotomy using polyacrylamide adhesive. The injury device was connected to the female luer loc using a male luer loc fitting. The device then induced injury by generating a 25 msec saline pressure pulse which was transmitted to the brain. This pressure pulse results in a transient deformation of brain tissue at the site of the craniotomy. The amplitude of the pressure pulse was measured using a pressure transducer. In our experience, a 2.5 atmosphere pressure pulse results in moderate injury. Throughout this procedure the rats remained anaesthetized. After confirming that the condition of the rat was stable, the luer loc fitting was removed, the craniotomy was sutured, the skin sutured and the animals removed from anaesthesia to permit recovery. Throughout all procedures, temperature of rats was maintained at 37°C using a heating pad. Rats usually woke within 15 minutes of injury and were exhibiting normal exploratory behaviour within 60 minutes.

### TBI outcome measures

Rats motor abilities were assessed by their ability to remain on a rotarod, according to established procedures [52]. Rats were trained on the rotarod for 5 days prior to injury unless they recorded the maximum time of 120 seconds for 2 consecutive days, in which case they were deemed trained. The final day of the training day was used as the pre-injury score. Rats were assessed at the same time each day for the 7 R/NIR-IT treatment days following injury. The Bilateral Asymmetry Test was used to assess sensory function. Forepaw latency was assessed according to established procedures [53], on both the ipsilateral and contralateral forepaws prior to induction of injury. Latency of both forepaws was then assessed on days 1, 3 and 7 post-injury. Due to the individual variability between animals, each rat's contralateral forepaw latency (forepaw which is affected) was compared to their ipsilateral forepaw latency to give a percentage of latency score.

Following perfusion fixation at 7 days after injury with 10% formalin, a 10 mm section of whole brain (Bregma; 1.0 mm to −9.0 mm AP) (Paxinos and Watson Rat brain stereotaxic atlas) was processed and embedded in paraffin. Serial sections were taken at every 250 um interval for the entire described brain region. Sections were stained with Haematoxylin and Eosin stain, scanned using the Hamamatsu Nanozoomer and viewed with the associated software. The area of the lesion was circumscribed using this software, with the area recorded for each section. The following equation was then used to determine lesion volume (mm^3^):  = ∑ (lesion volume of each section) * 0.25 (250 um): this method has been used routinely within our laboratory, modified from Corrigan et al., (2012) [54].

### SCI animals and surgery

Adult female Fischer rats (F344, 160–190 g) were bred at the Animal Resources Center (Animal Research Center, Murdoch, W.A.), housed under a standard 12 h light/dark cycle, fed wet and dry rat chow and water ad libitum. Each treatment group consisted of 7 or 8 rats, groups were control, 670 nm or 830 nm R/NIR-IT treated, total n = 22 for this model, all rats received the SCI. Moderate contusion SCI was performed as described in [55] and in brief as follows; rats were anaesthetised by i.p. injection of xylazine in combination with ketamine (50 mg/kg ketamine hydrochloride and 10 mg/kg xylazil hydrochloride, Troy Laboratories, NSW, Australia). Amacin ophthalmic eye ointment (Provet, Queensland, Australia) was applied after the rats were placed under anaesthetic. A longitudinal incision and partial laminectomy were performed at vertebral level T9–T10 to expose the underlying thoracic spinal cord without disrupting the dura. Rats were positioned on a surgical plate for spinal cord impact using flexible armatures and Adson forceps (spinal cord stabilizing forceps). All rats received a moderate contusion injury (200KDyne) using an Infinite Horizon impactor device on the dorsal surface of the exposed spinal cord. Rats were maintained in a temperature and humidity controlled chamber until recovery from anaesthetic. Rehydrating saline (2 mL) in conjunction with Buprenorphine Hydrochloride (Provet) (Temgesic, 0.01 ml/100 g body weight, 300 U/ml) and intramuscular injection of Benicillin (Provet) (0.02 mL/100 g body weight, 300 U/mL) was administered immediately after surgery and every 12 h thereafter to hydrate and act as an analgesic respectively. Bladders were manually expressed every 12 h until function was recovered and total volumes of given saline were adjusted for rat bladder size. Frequency of saline - Temgesic injections was reduced to every 24 h when animals were able to drink unassisted, and generally ceased around day 7–10 after SCI. 50 uL of Benicillin (Provet, 0.02 ml/100 g body weight, 300 U/mL, (150 mg/ml procaine penicillin, 150 mg/ml benzathine penicillin, 20 mg/ml procaine hydrochloride; Troy Laboratories Pty. Ltd., Glendenning, NSW, Australia)) was administered intramuscularly immediately after surgery and at 2, 4 and 6 days to prevent wound and bladder infections.

### SCI outcome measures

Functional assessments were performed on days 1, 2, 3, 7, 14, 21 and 28 following surgery and daily R/NIR-IT, and consisted of open field locomotion assessment, quantitative gait analysis (Ratwalk) and ladder walking. The Basso, Beattie, Bresnahan (BBB) locomotor rating scores were used to assess the range and type of open field spontaneous forward locomotion [56]. Rats were recorded in an open field for 4–5 minutes on days 7, 14, 21 and 28-post injury. Video recordings were made on the left, right and posterior views of the animal. Scoring was by 3 independent assessors blinded to animal treatments: assessors had to score animals within 1 point of each other or animals were re-assessed. Quantitative gait analysis using Ratwalk was performed at days 14, 21 and 28-post injury under red light, according to established procedures [57] and assessed using Ratwalk software. The stride length was obtained by measuring the distance (in pixels) between successive placements of an individual paw. Ladder walking was also assessed at days 14, 21 and 28 days post-injury. All rats were recorded walking the length of the ladder walk apparatus 3 times per session. Analysis was performed using video recordings, analysed frame-by-frame by 3 independent scorers. The foot faults for both the left and right limbs were recorded over the total distance travelled [58].

At day 28-post injury, animals were euthanized by lethal injection of sodium pentobarbitone (Provet; 50 mg/100 g) and perfused with approximately 200 mL of 1% (v/v) Heparin (Prover) in PBS, followed by approximately 250 mL of 4% (w/v) paraformaldehyde pH 7.2 (Sigma) in PBS. Spinal cords were dissected and cryosectioned longitudinally (40 µm). Sections were stained with toluidine blue for assessment of cyst size and for immunohistochemical analysis according to established procedures [55] (n = 4/group), using antibodies to: glial fibrillary acidic protein (GFAP) (1∶400, Dakopatts); β-III tubulin (1∶400, Covance); neuronal doublecortin (DcX) (1∶400, Novex); GAP43 (1∶400, Invitrogen) and activated macrophage ED1 (CD68) (1∶400, Serotec). Secondary antibodies were species specific AlexaFluor 555- and 488-conjugated antibodies (1∶500; Invitrogen).

Toluidine blue sections were scanned at high power magnification (40x) using a ScanScope digital slide scanner with Aperio Software. Analysis of the cyst area was carried out using the software Image-Pro Plus (Image-Pro version 3.0 for Windows, Media Cybemetics Image-Pro, Rockville MD) where the total area of cystic structures within ±5 mm (rostral-caudal axis) from the epicenter of the lesion was calculated as a percentage of total tissue. Immunohistochemical labelling was visualized in sections taken within the central part of the lesion (n = 4) in each treatment group (repeated 2x), and photographed using a Nikon Eclipse E800 microscope. All images for each outcome measure were captured at constant exposures and in a single session.

### Statistical analyses

All data were expressed as mean ± SEM: statistical analyses were conducted using SPSS statistical software (IBM) or PRISM (GraphPad Software). Data were analyzed using two-tailed Student's t test, one- or two-way ANOVA and Bonferroni Dunn or Dunnett's post-hoc tests as appropriate (F values, degrees of freedom (dF) and post-hoc test p values given). In the case of the open field locomotion test, the Kruskal Wallis post-hoc test for non-parametric data was used. Ladder walk analysis was performed using two-way ANOVAs comparing identical treatment groups over days 14, 21 and 28, and experimental conditions at each time point. Probabilities ≤0.05 were considered significant.

## Results

### Penetrance of light including R/NIR to a CNS injury site

**Figure 1:**
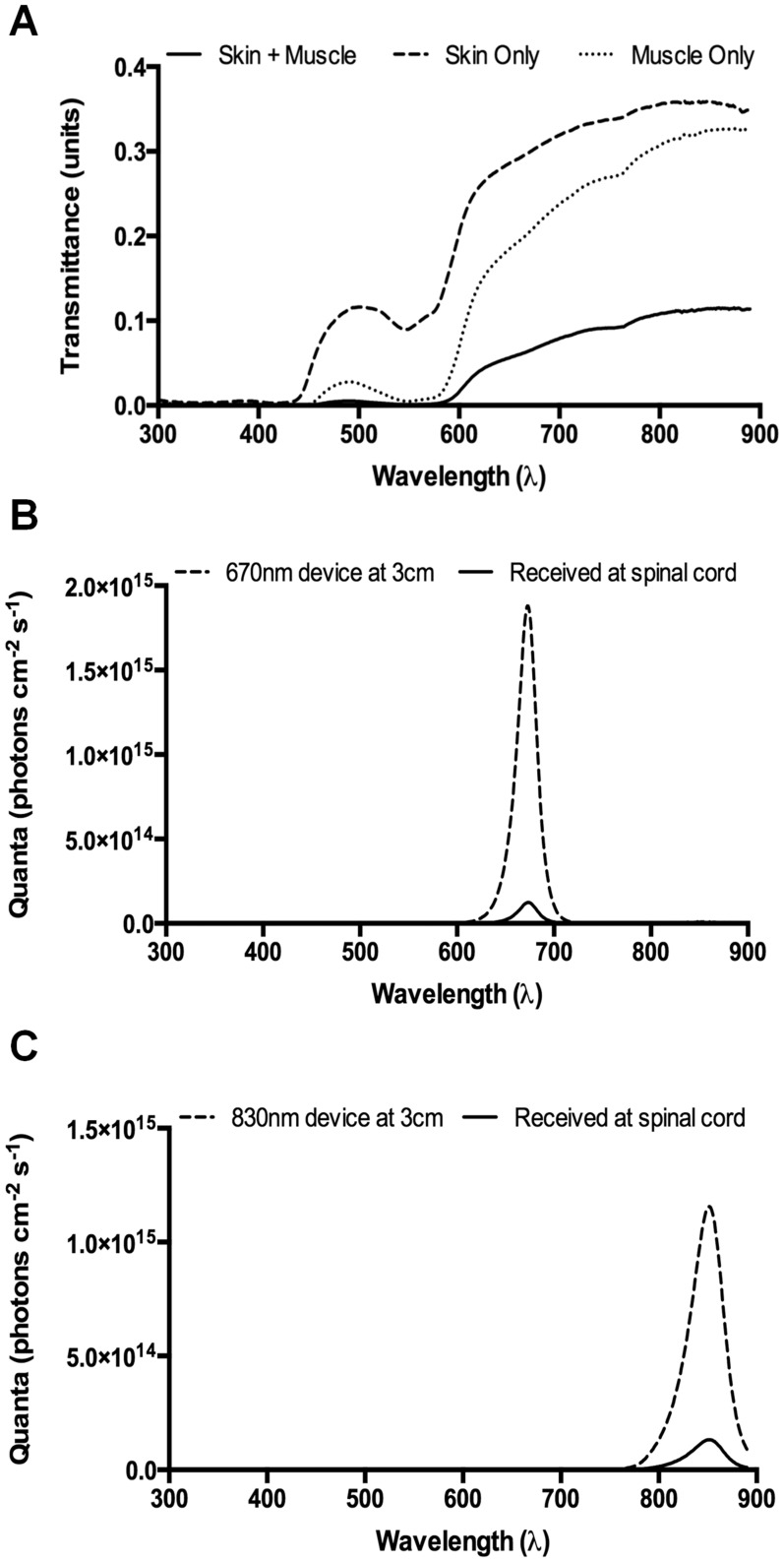
Penetrance of light through rat skin and muscle and irradiance delivered by the 670/NIR-IT LED arrays. (A) Spectral transmittance of light from 300 nm to 890 nm through skin (red), muscle (green), and skin plus muscle combined (blue), taken from the area overlying the SCI injury site in a Fischer rat cadaver (n = 1). Spectral irradiance (300–890 nm) received at the surface of the skin from the 670 nm (B) or 830 nm (C) LED arrays at a distance of 3 cm (blue lines) and calculated numbers of photons reaching the SCI injury site (red line).

The effect of wavelength of light on penetrance of R/NIR light through skin and/or muscle was assessed by measuring the spectral transmittance (300 nm to 890 nm) of the tissue overlying the spinal cord in a Fischer rat cadaver, allowing calculation of the irradiation doses received at the injury site in the SCI model. Transmittance was higher through skin than muscle, and was greatest between 750–890 nm, with a minor secondary peak at around 500 nm (Fig. 1A). Unlike lasers, LEDS are not monochromatic and instead are narrowband light sources that emit significant amounts of light either side of the specified emission peak (Fig. 1A, B). Thus, measured irradiances produced by the LED devices are integrated over the wavelength range 400–890 nm. The total irradiance of R/NIR-IT received at the surface of the skin, 3 cm from the emission surface of the 670 nm device, was 5.3×10^16^ photons cm^−2^ s^−1^ (peak irradiance at 670 nm was approximately 2×10^15^ photons cm^−2^ s^−1^) and this was calculated to drop to 3.5×10^15^ photons cm^−2^ s^−1^ (6.6%) at the SCI injury site, below the overlying skin and muscle (Fig. 1B). Similarly, the total irradiance of R/NIR-IT received at the surface of the skin, 3 cm from the surface of the 830 nm device, was 5.3×10^16^ photons cm^−2^ s^−1^ (peak irradiance at 830 nm was approximately 1.2×10^15^ photons cm^−2^ s^−1^) and this dropped to 6.0×10^15^ photons cm^−2^ s^−1^ (11.3%) at the SCI injury site (Fig. 1C).

### Effects of R/NIR-IT following partial optic nerve injury

**Figure 2:**
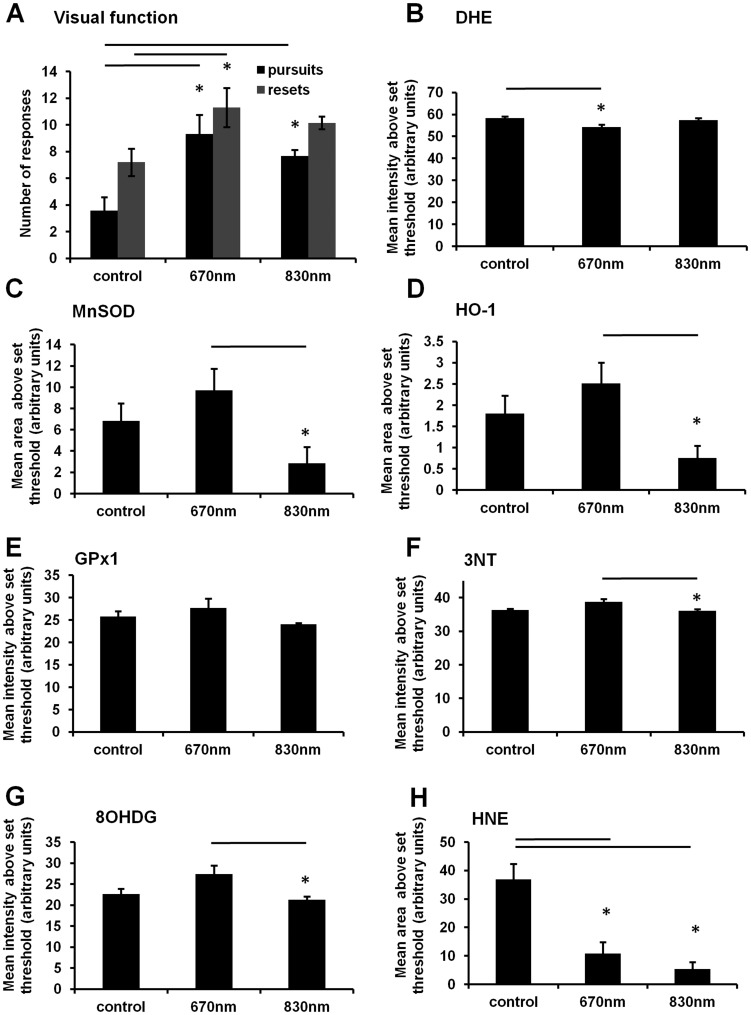
Visual behaviour, reactive species, antioxidant enzymes and indicators of oxidative damage 7 days following partial optic nerve transection and treatment with 670/NIR-IT compared to sham treated control. Number of responses in the optokinetic nystagmus visual reflex task (A) were quantified as smooth pursuits (black) or fast resets (grey), n = 12/group. Mean ± SEM DHE fluorescence (B), MnSOD (C), HO-1 (D), GPx1 (E), 3NT (F), 8OHDG (G) or HNE (H) immunoreactivity in ventral ON vulnerable to secondary degeneration were semi-quantified: *indicates significant differences (P≤0.05), n = 6/group.

Partial dorsal optic nerve transection leaves the remaining ventral optic nerve vulnerable to secondary degeneration, and provides a model to assess efficacy of therapeutic strategies to prevent spreading damage following injury to the CNS [59]. Analysis of functional behaviour following partial optic nerve transection and R/NIR-IT was assessed using the optokinetic nystagmus visual reflex at 7 days after injury. R/NIR-IT delivered at 670 nm led to a significantly increased number of smooth pursuits and fast reflexes compared to numbers of responses by injured, control animals (F = 12.8 and 3.9 respectively, dF = 2, P≤0.05), indicating improved visual function and confirming previous findings [22]. R/NIR-IT at 830 nm resulted in significantly more smooth pursuits compared to control (F = 12.8, dF = 2, P≤0.05), but there was no significant improvement in the number of fast resets (F = 3.9, dF = 2, P>0.05, Fig. 2A). There were no significant differences between either numbers of smooth pursuits or fast reset responses by rats treated with 670 nm R/NIR-IT, compared to rats treated with 830 nm R/NIR-IT (P>0.05) (Fig. 2A).

DHE fluoresces upon oxidation by a range of reactive species and was used to semi-quantify increases in ROS/RNS, in fresh frozen tissue sections. Note that data shown here and throughout are either mean areas or mean intensities above threshold, with choice of analysis dependent on the pattern of staining and the most consistent or pronounced increases observed with injury compared to normal optic nerve (unpublished data). In ventral optic nerve vulnerable to secondary degeneration, there was a small but significant decrease in the mean fluorescence intensity of DHE, when animals were treated with 670 nm R/NIR-IT (F = 5.3, dF = 2, P≤0.05) (Fig. 2B), indicating that 670 nm R/NIR-IT reduced ROS/RNS in CNS tissue vulnerable to spreading damage at 7 days. There was no effect of R/NIR-IT delivered at 830 nm on DHE fluorescence at this time (F = 5.3, dF = 2, P>0.05) (Fig. 2B).

The intensities of immunoreactivity of the antioxidant enzymes MnSOD, HO-1 and GPx1 were quantified in ventral optic nerve vulnerable to secondary degeneration at 7 days following injury. We observed no significant change in immunoreactivities when animals were treated with either 670 nm or 830 nm R/NIR-IT, compared to control at this time (F = 4.6, 4.5 and 1.7 respectively, dF = 2, P>0.05) (Figs. 2C–E), in contrast to our reported reductions in MnSOD following 670 nm R/NIR-IT at 1 day after injury[22]. MnSOD and HO-1 immunoreactivities in animals treated with 830 nm R/NIR-IT were significantly lower than in rats treated with 670 nm R/NIR-IT (F = 4.6 and 4.5 respectively, dF = 2, P≤0.05) (Figs. 2C–E). Similarly, there was no change in the immunoreactivity of the indicator of protein nitration 3NT or the indicator of oxidized DNA 8OHDG in ventral optic nerve, when rats were treated with either 670 nm or 830 nm R/NIR-IT, compared to control at 7 days (F = 5.0 and 4.6 respectively, dF = 2, P>0.05) (Figs. 2F, G). However, immunoreactivities of both 3NT and 8OHDG in rats treated with 830 nm R/NIR-IT were significantly lower than in rats treated with 670 nm R/NIR-IT (F = 5.0 and 4.6 respectively, dF = 2, P≤0.05) (Figs. 2F, G). Importantly, the immunoreactivity of the indicator of lipid oxidation HNE, was significantly reduced relative to control, when animals were treated with either 670 nm or 830 nm R/NIR-IT (F = 15.0, dF = 2, P≤0.05) (Fig. 2H), indicating reduced oxidative stress. There was no significant difference in HNE immunoreactivity between the two wavelengths of R/NIR-IT at this time (F = 15.0, dF = 2, P>0.05) (Fig. 2H).

### Effects of R/NIR-IT on light-induced retinal degeneration

**Figure 3:**
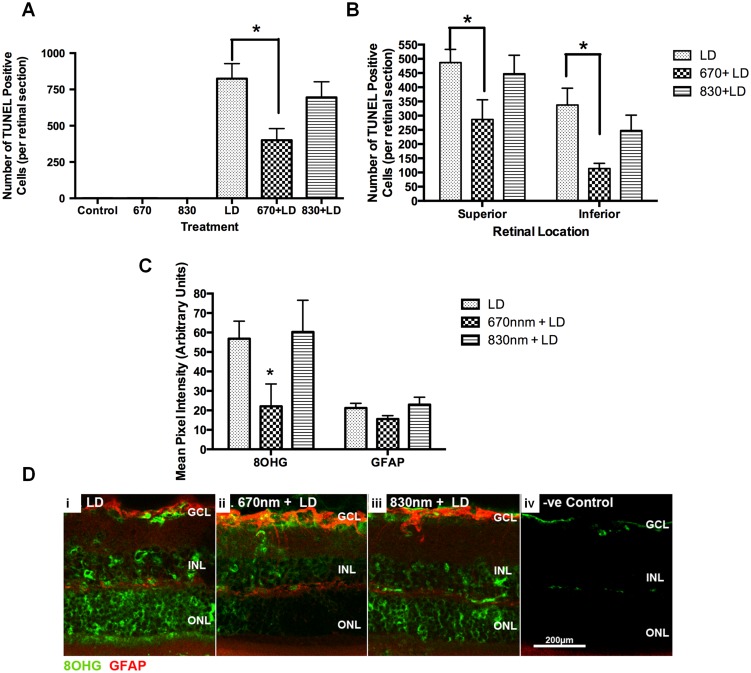
Number of Tunel+ cells, 8OHDG and GFAP immunoreactivity following pre-treatment with 670 nm or 830 nm R/NIR-IT or sham treatment and light induced retinal degeneration, compared to uninjured animals. Mean ± SEM total number of Tunel+ cells/retinal section (A) and number of Tunel+ cells in superior and inferior retina (B) were quantified. Mean ± SEM 8OHDG and GFAP immunoreactivities in retinal sections were semi-quantified (C): *indicates significantly different from injured control (P≤0.05), n = 6/group. Representative images of 8OHDG (green) and GFAP (red) immunofluorescence from control and R/NIR-IT treated animals are shown (D) (scale  = 200 µm).

Light damage induces cell death, indicated by large numbers of TUNEL positive (+) cells in rat retina from as early as 6 hours after injury, particularly in the photoreceptor layers, as described previously [60], [61]. We detected no effect of pre-treatment with 830 nm R/NIR-IT on numbers of TUNEL+ cells relative to controls (F = 1.417, dF = 5, P>0.05) (Fig 3A). In contrast, we observed a significant decrease in the total number of TUNEL^+^ photoreceptors in the outer nuclear layer of LD retinae pre-treated with 670 nm R/NIR-IT compared with controls, confirming previous findings (F = 1.678, dF = 5, P≤0.05) [62], [63]. The lower numbers of TUNEL+ cells in retinas treated with 670 nm R/NIR-IT were significant in samples taken from both superior retina and inferior retina (F = 3.423, dF = 2, P≤0.05) (Fig. 3B). Immunoreactivity for 8OHDG, indicative of oxidised DNA and therefore oxidative stress, was significantly reduced in LD retinae treated with 670 nm R/NIR-IT (F = 1.632, dF = 4, P≤0.05), but not those treated with 830 nm R/NIR-IT (F = 3.267, dF = 4, P>0.05) (Fig. 3C). While there was a trend towards reduced GFAP immunoreactivity following treatment of light damaged retinae with 670 nm R/NIR-IT, this was not significant (F = 1.867 dF = 4, P>0.05) (Figs. 3C, D).

### Effects of R/NIR-IT following TBI

**Figure 4:**
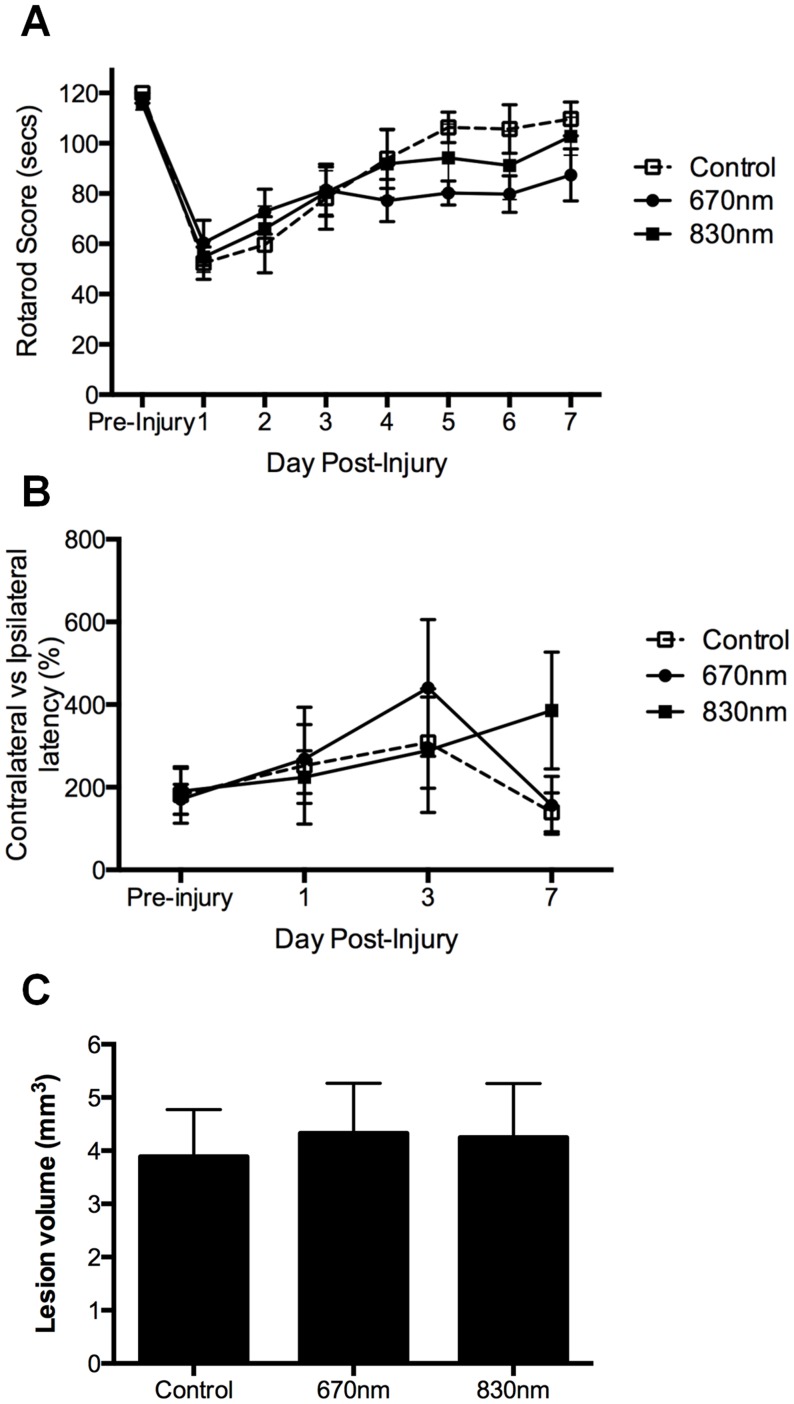
Motor and sensory function as well as lesion size following fluid percussion TBI and treatment with 670/NIR-IT compared to sham treated control. Mean ± SEM length of time animals remained on a rotarod (A), and contralateral vs ipsilateral latency (B) were quantified as measures of motor and sensory function respectively over the 7 days following TBI. Lesion volumes were also quantified at 7 days following injury (C): there were no significant differences relative to control at each time point (P>0.05), n = 5 or 6/group.

The fluid percussion model was used to assess efficacy of 670 nm and 830 nm R/NIR-IT at improving motor and sensory outcomes as well as decreasing lesion volume following TBI. Motor ability of injured rats was assessed daily by quantifying the length of time rats could remain on a rotarod. As expected [64], there was a substantial decrease in rotarod score relative to pre-injury in control animals (significant for the first 3 days following injury, P≤0.05), which gradually improved during the 7 days following injury (Fig. 4A). R/NIR-IT at 670 nm or 830 nm did not affect motor outcomes during the 7 days following TBI, relative to control (F = 0.5, dF = 2, P>0.05) (Fig. 4A). Sensory ability was assessed using the bilateral asymmetry test as has been described [53], at 1, 3 and 7 days following injury, compared to pre-injury performance. However, somewhat unexpectedly, there was no significant difference in contralateral *vs* ipsilateral latency in control (sham treated) rats when comparing pre- and post-injury outcomes for the first 7 days after injury (F = 0.4, dF = 2, P>0.05) (Fig 4B). Sensory abilities in rats treated with R/NIR-IT at 670 nm or 830 nm were not different to control rats or pre-injury levels (F = 0.4, dF = 2, P>0.05) (Fig. 4B). Lesion volume at 7 days following TBI was also not altered by R/NIR-IT at either 670 nm or 830 nm (F = 0.1, dF = 2, P>0.05) (Fig. 4C).

### Effects of R/NIR-IT following SCI

**Figure 5:**
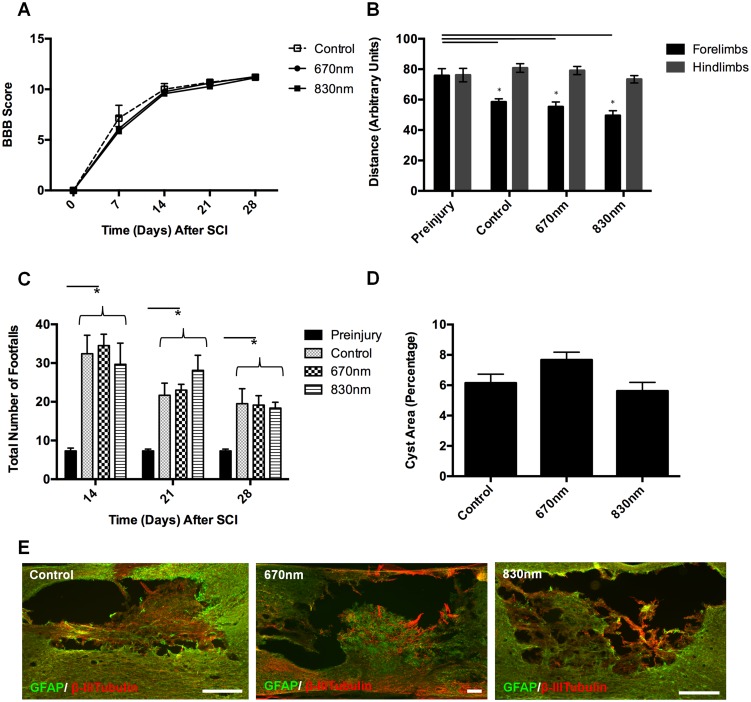
Analysis of functional recovery and lesion size following SCI and treatment with 670/NIR-IT compared to sham treated control. Functional recovery was quantified for 28 days using BBB scores (A), Ratwalk gait analysis (B) and ladder walking (C) (n = 7 or 8/group). Semi-quantification of lesion size from toluidine blue stained sections (D), and representative images of GFAP and β-III tubulin immunoreactivity (E) are shown at 28 days following SCI (n = 4/group), scale  = 400 µm. Data are mean ± SEM, * indicates significant differences from pre-injury (P≤0.05).

Analysis of functional recovery following moderate contusion SCI and R/NIR-IT was conducted, assessing open field locomotion (BBB), quantitative gait analysis (Ratwalk) and ladder walking. Rats were scored for their performance on the BBB test at 7, 14, 21 and 28 days following SCI [56]. All animals exhibited a steep recovery from day 0 to 7 and were able to weight support by day 14 (BBB score of 9). At day 21, the scores began to plateau and the average score across all groups was 10. At day 28 the average score for all groups was 11 - characterised by frequent to consistent weight-supported plantar steps and no forelimb - hindlimb coordination. There were no significant differences in BBB scores of rats treated with either wavelength of R/NIR-IT compared to control rats (Fig. 5A, F = 0.5, dF = 2, P>0.05). Although no significance was recorded, it is important to note that even though the applied Kruskal-Wallis test is an analysis of variance for non-parametric data, the BBB scale is neither linear nor exponential. Higher BBB values represent a score comprised of increasing numbers of variables that are often unrelated to previous (lower) scores. Careful interpretation should always be exercised when equating biological significance with statistical significance in such cases [65].

The gait of rats in the treatment groups was analysed using Ratwalk computer software at days 14, 21 and 28 following SCI and R/NIR-IT, and stride lengths were quantified. Data generated from animals pre-injury were used to illustrate ‘normal’ locomotor patterns (baseline) and as a reference point for comparison to R/NIR-IT treated rats following SCI. Data generated from left and right limbs of the fore-and hindquarters were compared and no significant differences were found (P>0.05). Therefore, data generated from left and right forelimbs and left and right hindlimbs were averaged and referred to as forelimbs and hindlimbs respectively. The stride length of forelimbs following SCI was significantly reduced in all treatment groups and time-points following injury, compared to scores generated from pre-injury animals (Fig. 5B, 28 day data shown, F = 15.4, dF = 3, P≤0.05). This indicated that there was some compensatory change in forelimb movement following SCI, most likely as hindlimb function is impaired. The animals use their forelimbs to propel their body forward, a movement which would not otherwise be possible if the stride length of the forelimb remained at its original pre-injury distance. However, there were no significant differences in forelimb stride length of rats treated with either wavelength of R/NIR-IT compared to control sham-treated rats (Fig. 5B, F = 15.4, dF = 3, P>0.05). The stride length of the hindlimbs did not change with SCI (Fig. 5B, F = 1.6, dF = 3, P>0.05).

There were significantly more hindlimb missteps following SCI at each time point assessed, for both R/NIR-IT treated and control groups (F = 3.4, dF = 6, Fig. 5C, P≤0.05), compared to pre-injury performance. The total number of missteps at 21 and 28 days after injury, for the control and 670 nm R/NIR-IT groups, was significantly lower than the number of missteps at 14 days (F = 3.4, dF = 6, Fig. 5C, P≤0.05), whereas treatment with 830 nm R/NIR-IT only resulted in significant decreases in the total number of missteps at 28 days, compared to 14 days (F = 3.4, dF = 6, Fig. 5C, P≤0.05). However, there were no significant differences between R/NIR-IT treatment and control groups at any of the time points assessed (F = 3.4, dF = 6, P>0.05).

Cyst size and morphological parameters, including the amount of degenerative tissue immediately surrounding the cyst, were assessed in toluidine blue stained sections and using immunohistochemical assessments of GFAP, β-III tubulin, DcX and GAP43 immunoreactivity at 28 days following SCI. The borders of larger cysts stained with toluidine blue were well defined, whereas smaller cysts were present within spared tissues to varying degrees in all R/NIR-IT treatment and control groups. Semi-quantitative analysis of cyst size revealed no significant differences between 670 nm or 830 nm R/NIR-IT and control groups (Fig. 5D, F = 3.9, dF = 2, P>0.05). In all groups GFAP immunoreactivity was present around the lesion site and occasionally appeared within “spared” bridges of tissue within the lesion site, whilst β-III tubulin was present both in and around the lesions site (Fig. 5E). In some sections β-III tubulin positive fibres were seen to traverse across the lesion site within spared tissue, but this did not appear to be specific for any particular group, and was therefore not quantitated. Small numbers of DcX and GAP43 positive fibres penetrated into tissue located within the cyst region, although these tended to be located immediately at the periphery of the cyst walls, and the number of fibres did not appear to differ between groups (data not shown). Infiltrating macrophage numbers (ED1+) remained high within and around the lesion area and projected several mm beyond the lesion epicentre, with no effect of R/NIR-IT observed (data not shown).

## Discussion

R/NIR-IT has the potential to be a clinically relevant, cost-effective and easy-to-administer treatment to reduce oxidative stress and preserve function following traumatic injury. However, R/NIR-IT has not been widely adopted in clinical practice for CNS injury, in part due to the large range of treatment fluences, irradiation time, wavelengths and devices cited in the literature making comparisons between studies difficult. As such, a consensus paradigm for treatment has not yet been established[48]. Here we report the outcomes of a multi-centre comparative study which shows that delivery of a defined number of photons at the surface of the skin overlying a CNS injury has differential effects on function and neuroprotection (as indicated by Tunel staining), depending upon the wavelength used to deliver the R/NIR-IT and the injury type.

Our study design allows the first direct comparison of efficacy of 670 nm and 830 nm R/NIR-IT, delivered by LED array, in four models of CNS injury. We demonstrate that the most effective wavelength for treatment depends upon the type of injury. Specifically, 830 nm R/NIR-IT is beneficial following partial optic nerve transection but not following light-induced retinal degeneration. Given the greater penetrance of R/NIR-IT to the retina than to the optic nerve, and our demonstration of efficacy of 670 nm R/NIR-IT for treatment of retinal degeneration, our data indicate that the nature of the tissue being treated dictates the potential efficacy of different wavelengths of irradiation. Further, our study design allows us to demonstrate that wavelengths and dosages of R/NIR-IT that are effective in one model of CNS injury (e.g. partial optic nerve transection) are not necessarily effective in another. The demonstrated penetrance of R/NIR-IT to the site of SCI in the current study was greater than that to the optic nerve injury site, yet there was no positive effects on function following SCI [22]. We conclude therefore that delivery of equivalent or greater dosages of R/NIR-IT does not guarantee beneficial effects across different CNS injury models.

670 nm R/NIR-IT delivered by LED was generally more effective at improving function or protecting neurons than 830 nm R/NIR-IT, but this only applied in models involving the visual system. The greater efficacy of 670 nm R/NIR-IT was apparent despite a greater percentage of delivered photons of 830 nm than 670 nm R/NIR-IT penetrating through skin and muscle, leading to a likely greater dose of 830 nm R/NIR-IT at the injury site. Neither 670 nm nor 830 nm R/NIR-IT delivered by LED array were effective at improving outcomes following TBI or SCI at the dosages and time points assessed. Our data could be interpreted to indicate that R/NIR-IT delivered by LED array is more effective in the visual system than in other CNS injuries, but it is important to note that the beneficial effects observed in the retinal degeneration model may have been enhanced due to the pre-treatment paradigm employed. Nevertheless, our study indicates that effective R/NIR-IT treatment parameters including wavelength and dosage do not necessarily apply in different CNS injuries. Efficacy does not appear to be directly proportional to the numbers of photons reaching the injury site, but may instead be a function of the number of photons absorbed by the photoacceptor responsible for any beneficial effects. For example, the absorbance of cytochrome *c* oxidase is much greater at 670 nm than 830 nm [66], which may explain why the 670 nm light was more effective, despite the injury site receiving a lower fluence at this wavelength compared to 830 nm. Furthermore, analysis of the absorption spectra of cytochrome *c* oxidase within cell monolayers indicates that the enzyme is oxidised by irradiation at 670 nm, whereas it is reduced at 830 nm [26], perhaps also contributing to differential efficacies.

To date, few studies have compared R/NIR-IT in CNS at two or more wavelengths. Wong-Riley et al., 2005 assessed ATP production by visual cortex neurons treated with 670, 728, 770, 830 or 880 nm R/NIR-IT (*via* LED) emitting 4 J cm^−2^ over 80 sec *in vitro*, demonstrating a positive correlation with the known absorption spectra of cytochrome *c* oxidase, the postulated photoacceptor for R/NIR-IT [29]. Comparison of efficacy of 665, 730, 810 or 980 nm R/NIR-IT delivered by laser emitting 36 J cm^−2^ over 4 min *in vivo* following acute contusion TBI resulted in significant improvements in Neurological Severity Scores in animals treated with 665 nm and 810 nm R/NIR-IT [45]. Although these studies did not appear to standardise the dosages for the numbers of photons delivered at different wavelengths, our choices of wavelengths to assess in the current study were based on those comparative assessments.

The penetrance of irradiation at a given wavelength is determined by the optical properties of the tissue being treated. Irradiation between 600 and 1000 nm has better penetration because of reduced absorption at these wavelengths [48]. We demonstrate here that while the 830 nm R/NIR-IT array delivered an equal quantal dose to the surface of the skin (5.33×10^16^ photons cm^−2^ s^−1^), the number of photons received at the SCI injury site was greater due to the increased penetrance of the longer wavelength R/NIR-IT through tissue (6.0×10^15^ photons cm^−2^ s^−1^ for the 830 nm device compared to 3.5×10^15^ photons cm^−2^ s^−1^ for the 670 nm device). To our knowledge, two published studies have quantified the penetrance of R/NIR irradiation in animal specimens. Byrnes et al., 2005 used power transmission and spectrophotometric analyses to show that 6% (9 mW) of the radiant power of an 810 nm laser irradiation with a nominal power output of 150 mW, was transferred from the dorsal surface of the skin to the ventral side of the spinal cord [16]. We have also previously shown that irradiation directed at the dorsal surface of the head of the rat (R/NIR irradiance 252 W m^−2^ 550–750 nm, WARP10, Quantum Devices), resulted in 0.1% (0.3 W m^−2^) reaching the ventral surface of the braincase and 0.7% (1.75 W m^−2^ or 0.32 J cm^−2^) reaching the ventral surface of the optic nerve[22]. These light levels achieved therapeutic benefits and were significantly lower than the doses used in the superficial wound healing studies (8 J cm^−2^) [67] implying that relatively low doses of light can have therapeutic benefits.

Nevertheless, delivery of doses of 28.4 J cm^−2^ of 670 nm R/NIR-IT or 22.6 J cm^−2^ of 830 nm R/NIR-IT (equal quantal doses) in the current study, equating to 1.9 J cm^−2^ and 2.6 J cm^−2^ respectively at the SCI injury site, did not result in improvements following SCI. The doses we administered fall within the lower end of the range of doses of R/NIR-IT delivered by laser and shown to have beneficial effects in SCI (9.6–250 J cm^−2^ [68], [69] emitted from the treatment devices). Importantly however, previous studies demonstrating functional improvements with R/NIR-IT following SCI utilised a substantially higher dosage of 1589 J cm^−2^ day^−1^ [16], [21]. It remains a possibility that beneficial effects of R/NIR-IT delivered by laser for SCI are dependent on the coherence, narrow beam profile or some other physical aspect of laser light. However, a comprehensive assessment of the effects of an increased dosage of R/NIR-IT delivered using LED arrays would be necessary before such a conclusion could be drawn. Alternatively, the time points at which efficacy of R/NIR-IT for SCI were assessed may have been too early after injury to reveal improvements, although efficacy has been demonstrated at 21 days in other SCI studies [21], [69], indicating that we conducted our assessments within an appropriate time-scale. Further, it is possible that the handling of animals required for the 30 minute treatments using the LED arrays, increased stress levels such that improvements following SCI and/or TBI were masked. However, given that efficacy was demonstrated in visual system models where animals were handled similarly, we consider this to be unlikely. It is perhaps more likely that the degree of inflammatory involvement, vascular changes, cyst formation, oedema and myelination [70]–[72] are likely to alter penetrance of the R/NIR-IT [48], [73] and may explain the lack of efficacy following SCI at the current comparatively low dosage. The moderate contusion SCI may be too severe an injury to enable detection of modest improvements at the doses of R/NIR-IT employed in the current study. Other spinal cord injury types such as hemisection, or injuries targeting limited demyelination of specific tracts (e.g. corticospinal), may prove more effective at revealing modest improvements.

The doses of R/NIR-IT delivered by LED in the current study also did not result in significant improvements in the tested model of TBI. Previous assessments of efficacy of R/NIR-IT for TBI in rodents have predominantly used lasers and have delivered doses ranging from 1.2–268 J cm^−2^ [74]. Improved neurobehavioural function and reduced lesion size have been reported at four weeks after acute, relatively severe cortical impactor injuries [45], [75], [76]. However, approximately equivalent dosages of R/NIR-IT, also delivered by laser, were not effective at improving motor function or lesion volume following a less severe cortical piston TBI, assessed at 1 week after injury [77]. Similarly, the lack of positive effect may be due to the relatively mild nature of the focal TBI delivered in the current study, resulting in lack of a sufficient window of opportunity in which to detect improvements. Spontaneous return of function was observed by seven days after injury in our model, rendering longer term assessments irrelevant in this model. However, beneficial effects on function in TBI may require a longer time scale to become apparent. Importantly, our demonstration of lack of efficacy highlights the need to optimise R/NIR-IT with regard to injury severity.

Our demonstration of greater efficacy of 670 nm than 830 nm R/NIR-IT in models of injury to the visual system was unexpected, given that more photons are likely to have been delivered to the injury site with the 830 nm irradiation (given calculations for the SCI injury site). It is possible that there is a biphasic effect, with higher numbers of photons less effective than lower numbers [41], [78], such that dosage needs to be carefully titrated for each CNS injury model to ensure the most effective treatment protocol. Importantly however, the cellular targets in the various CNS injury models are different. 670 nm R/NIR-IT pre-treatment protected photoreceptors and reduced oxidative damage to DNA in the retinal degeneration model; 830 nm R/NIR-IT pre-treatment had no effect, with the caveat of a lower dosage of irradiation (shorter time of exposure) delivered in this model. It is possible that 670 nm R/NIR-IT readily penetrates to the retina and so the dosage received is greater and thus, more effective, than the 830 nm R/NIR-IT. In contrast, in the optic nerve damage model, both 670 and 830 nm R/NIR-IT reduced HNE immunoreactivity, indicative of lipid oxidation, in lipid rich ventral optic nerve white matter vulnerable to secondary degeneration. Furthermore, both wavelengths resulted in improved visual function in this model. The types of cells that are damaged or vulnerable in the different CNS injuries may be critical in determining the number of photons required to modulate disrupted cellular functions such as oxidative metabolism, given differential sensitivities to oxidative stress of different cell types [79], [80]. Whether it is the neuronal somata or the lipid-rich myelinated axons as opposed to unmyelinated axons that are vulnerable may also influence efficacy of R/NIR-IT.

The outcomes of the study indicate that comprehensive optimisation of the dosages, wavelengths and delivery mechanisms (laser vs LED) of R/NIR-IT appear to be required in each model of CNS injury, to provide an indication of effective treatment protocols. These must then be confirmed and refined in human clinical trials that draw upon these rationally designed pre-clinical studies. Only then will we know whether R/NIR-IT can provide an effective, cheap and convenient first-line treatment strategy for CNS injury.
